# Antioxidant and hepatoprotective effect of *Garcinia indica* fruit rind in ethanol-induced hepatic damage in rodents

**DOI:** 10.2478/v10102-012-0034-1

**Published:** 2012-12

**Authors:** Vandana Panda, Hardik Ashar, Sudhamani Srinath

**Affiliations:** 1 Department of Pharmacology & Toxicology, Prin. K. M. Kundnani College of Pharmacy, Rambhau Salgaonkar Marg, Cuffe Parade, Colaba, Mumbai, India; 2 Department of Pathology, Dr. D.Y. Patil Medical College, Nerul, Navi Mumbai, India

**Keywords:** *Garcinia indica*, ethanol, hepatoprotective, antioxidant activity

## Abstract

The protective effects of aqueous extracts of the fruit rind of *Garcinia indica* (GIE) on ethanol-induced hepatotoxicity and the probable mechanisms involved in this protection were investigated in rats. Liver damage was induced in rats by administering ethanol (5 g/kg, 20% w/v p.o.) once daily for 21 days. GIE at 400 mg/kg and 800 mg/kg and the reference drug silymarin (200 mg/kg) were administered orally for 28 days to ethanol treated rats, this treatment beginning 7 days prior to the commencement of ethanol administration. Levels of marker enzymes (aspartate aminotransferase (AST), alanine aminotransferase (ALT) and alkaline phosphatase (ALP)), triglyceride (sTG), albumin (Alb) and total protein (TP) were evaluated in serum. Antioxidant parameters (reduced glutathione (GSH), superoxide dismutase (SOD), catalase (CAT), glutathione peroxidase (GPx) and glutathione reductase (GR)), hepatic triglycerides (hTG) and the lipid peroxidation marker malondialdehyde (MDA) were determined in liver. GIE and silymarin elicited significant hepatoprotective activity by attenuating the ethanol–elevated levels of AST, ALT, ALP, sTG, hTG and MDA and restored the ethanol-depleted levels of GSH, SOD, CAT, GPx, GR, Alb and TP. GIE 800 mg/kg demonstrated greater hepatoprotection than GIE 400 mg/kg. The present findings indicate that hepatoprotective effects of GIE in ethanol-induced oxidative damage may be due to an augmentation of the endogenous antioxidants and inhibition of lipid peroxidation in liver.

## Introduction

“*Leave your drugs in the chemist's pot if you can heal the patient with food*”, the famous words of Hippocrates, the father of medicine have come of age. Recently, with the advent of functional foods and nutraceuticals, the focus has shifted to evaluation of dietary components, including fruits and vegetables as possible therapeutic interventions.

*Garcinia indica* Choisy (Family: *Clusiaceae/Guttiferae*), a slender evergreen tree is endemic to the west coast of India (Khare, 2007). It has many culinary, pharmaceutical and industrial uses. The dried rind known as “kokum” is an Indian spice and condiment used in many parts of the country for making several “curry” preparations. Syrup made from the fruits is a healthy soft drink used during summer to relieve sun stroke. Many therapeutic effects of the fruit have been described in Ayurveda, which include its usefulness as an infusion and in skin ailments such as rashes caused by allergies; in treatment of burns, scalds and chaffed skin; as a remedy for dysentery and mucous diarrhea; as an appetizer and a good liver tonic; as a cardiotonic and for bleeding, piles, tumors and heart diseases (Shastri, 1956).

One of the ingredients of kokum, hydroxycitric acid (HCA), has been patented for use as a hypocholesterolemic agent (Jena *et al.*, 2002). Another constituent from *G. indica*, Garcinol, a polyisoprenylated benzophenone has been reported to be an antioxidant (Devasagayam *et al.*, 2006; Krishnamurthy & Sampathu, 1988), a glycation inhibitor (Yamaguchi *et al.*, 2000a), and an antiulcer agent (Yamaguchi *et al.*, 2000b). It possesses a strong growth-inhibitory effect against human leukemia HL-60 cells (Pan *et al.*, 2001). Garcinol also shows antibacterial activity against Methicillin-resistant *Staphylococcus aureus,* comparable with that of the antibiotic Vancomycin (Rukachaisirikul *et al.*, 2005; Iinuma *et al.*, 1996). It is a natural histone acetylase transferase inhibitor both *in vitro* and *in vivo*, suggesting its implication in a wide variety of diseases like cancer and AIDS (Balasubramanyam *et al.*, 2004; Mantelingu *et al.*, 2007). Apart from HCA and garcinol, kokum contains other compounds like citric acid, malic acid, polyphenols, anthocyanin pigments and ascorbic acid with potent antioxidant properties (Padhye *et al.*, 2009). It is well known that heavy consumption of alcohol is associated with liver damage. Experimental animals exposed to two-carbon alcohol display biochemical signs of hepatotoxicity and oxidative damage, suggesting a possible role of free radicals in causing some of the toxic effects of alcohol (Albano *et al.*, 1999). The close relation between ethanol (ETOH) and liver damage is mainly due to the fact that about 80% of ingested alcohol is metabolized in the liver. Ethanol is metabolized to the cytotoxic acetaldehyde by the enzyme alcohol dehydrogenase in the liver. Acetaldehyde is oxidized to acetate by aldehyde oxidase or xanthine oxidase, giving rise to reactive oxygen species (ROS) via cytochrome P450 2E1 (CYP 2E1) (Tuma & Casey, 2003). Excessive alcohol consumption not only enhances ROS generation, but also depletes antioxidants, thus creating a state of oxidative stress leading to severe liver injury (Wu & Cederbaum, 2003).

Traditionally, the syrup of *G. indica* has been administered to alcohol intoxicated individuals for liver protection. Aqueous extracts of *G. indica* have been reported to possess potent antioxidant, free radical scavenging and anti-lipid peroxidative activities (Devasagayam *et al.*, 2006). Promising results of our previous study, the hepatoprotective effects of *G. indica* in CCl_4_-induced hepatotoxicity in rats, encouraged us to further explore this area (Panda & Ashar, 2012). With this background, the present study was carried out to investigate the hepatoprotective effect and a possible underlying antioxidant activity of aqueous extracts of the fruit rind of *G. indica* by assaying various marker enzymes, antioxidant enzymes and GSH in chronically ethanol fed rats.

## Material and methods

### Plant material

The fruit rind of *G. indica* was collected from the Konkan region of Maharashtra and air dried under shade, powdered mechanically and stored in an air tight container. The powder was extracted using soxhlet apparatus and water as solvent and stored in a refrigerator for further use. The plant was authenticated at the Blatter Herbarium, St. Xavier's College, Mumbai, after matching with the existing specimen (accession no. 03587).

### Drugs and chemicals

Silymarin was a gift from Ranbaxy Laboratories, Delhi, India. Thiobarbituric acid (TBA), reduced glutathione, oxidized glutathione and nicotinamide adenine dinucleotide phosphate (NADPH) were obtained from Himedia Laboratories, Mumbai, India. Epinephrine and 5, 5^’^-dithiobis (2-nitrobenzoic acid) – (DTNB) were purchased from Sigma Chemical Co., St Louis, MO, USA. All other chemicals were obtained from local sources and were of analytical grade.

### Experimental animals

Wistar albino rats (150–200 g) of either sex were used. They were housed in clean polypropylene cages under standard conditions of humidity (50±5%), temperature (25±2°C) and light (12 h light/12 h dark cycle) and fed a standard diet (Amrut laboratory animal feed, Pune, India) and water *ad libitum*. All animals were handled with humane care. Experimental protocols were reviewed and approved by the Institutional Animal Ethics Committee (Animal House Registration No.25/1999/CPCSEA) and conform to the Indian National Science Academy Guidelines for the Use and Care of Experimental Animals in Research.

### Preparation of test and reference drug solutions

*Garcinia indica* extract (GIE) was dissolved in distilled water and the aqueous solution was used. Silymarin was suspended in an aqueous solution of 1% carboxymethyl cellulose and administered.

### Experimental procedure

(Faremi et al., 2008; Pramyothin et al., 2007; Naik & Panda, 2007)

Animals, after acclimatization (6–7 days) in the animal quarters, were randomly divided into five groups of six animals each and treated in the following manner:

Group I served as normal control and received distilled water orally once daily for 28 days.

Group II served as toxicant control and received ethanol (5 g/kg, 20% w/v p.o.) once daily for 21 days from day 8 to day 28.

Group III, termed GIE400, received GIE (400 mg/kg, p.o.) daily for 28 days and ethanol (5 g/kg, 20% w/v p.o.) daily for 21 days from day 8 to day 28.

Group IV, termed GIE800, received GIE (800 mg/kg, p.o.) daily for 28 days and ethanol (5 g/kg, 20% w/v p.o.) daily for 21 days from day 8 to day 28.

Group V received silymarin (200 mg/kg, p.o.) for 28 days and ethanol (5 g/kg, 20% w/v p.o.) daily for 21 days from day 8 to day 28.

All the animals were humanely sacrificed using ether, 24 h after the last dose administration. Blood (3–4 mL) was collected by cardiac puncture under light ether anesthesia and allowed to clot for 30 min at room temperature. The serum was separated by centrifugation in an pathological centrifuge at 1,200× g at 30 °C for 15 min, and used for the determination of marker enzymes (aspartate aminotransferase (AST), alanine aminotransferase (ALT) and alkaline phosphatase (ALP)), serum triglyceride (sTG), albumin (Alb) and total protein (TP). The livers were dissected immediately, washed with ice-cold saline and divided into two equal parts. One part was used to prepare a 10% (w/v) homogenate in 1.15% KCl. An aliquot was used for the determination of lipid peroxidation (LPO). The homogenates were centrifuged in refrigerated tabletop centrifuge at 7,000×g for 10 min at 4°C and the supernatants were used for the assays of triglyceride (hTG), GSH, SOD, CAT, GPx and GR. The remaining part of the liver was fixed in 10% (w/v) buffered formalin and used for histological studies.

### Marker enzyme assays

The liver marker enzymes AST, ALT and ALP were assayed in serum using standard kits supplied from Span Diagnostics (Surat, India). The results were expressed as IU/L.

### Protein determination

The levels of TP and Alb were determined in the serum of experimental animals by using the Lowry method and the bromocresol green method, respectively (Lowry *et al.*, 1951; Webster *et al.*, 1974). Kits purchased from Span Diagnostics (Surat, India) were used for Alb assay.

### Triglyceride determination

Triglyceride levels were determined in the serum and tissue homogenate of experimental animals by using kits purchased from Biolab Diagnostics Ltd. (Mumbai, India)

### Lipid peroxidation

The quantitative determination of LPO was performed by measuring the concentration of thiobarbituric acid reactive substances (TBARS) in the liver using the method of Ohkawa (Ohkawa *et al.*, 1979). The amount of malondialdehyde (MDA) formed was quantified by the reaction with TBA and used as an index of lipid peroxidation. The results were expressed as nanomole of MDA per gram of wet tissue using molar extinction coefficient of the chromophore (1.56 × 10^–5^/M/cm) and 1,1,3,3-tetraethoxypropane as standard.

### Glutathione determination

GSH was assessed in the liver homogenate using DTNB by the method of Ellman (Ellman, 1959). The absorbance was read at 412 nm and the results were expressed as micromole per gram of wet tissue.

### Antioxidant enzyme assays in liver homogenate

SOD was assayed by the method of Sun and Zigman in which the activity of SOD was inversely proportional to the concentration of its oxidation product adrenochrome, which was measured spectrophotometrically at 320 nm (Sun & Zigman, 1978). One unit of SOD activity is defined as enzyme concentration required to inhibit the rate of auto-oxidation of epinephrine by 50% in 1 min at pH 10.

CAT was determined by the method of Clairborne, which is a quantitative spectroscopic method developed for following the breakdown of H_2_O_2_ at 240nm in unit time for routine studies of CAT kinetics (Clairborne, 1985).

GPx determination was carried out using the method of Rotruck *et al.* (1973) which makes use of the following reaction:

H_2_O_2_ + 2GSH → 2H_2_O + GSSG (oxidized glutathione)

GPx in the tissue homogenate oxidizes glutathione and simultaneously H_2_O_2_ is reduced to water. This reaction is arrested at 10 min using trichloroacetic acid and the remaining glutathione is reacted with DTNB solution to result in a colored compound, which is measured spectrophotometrically at 420 nm (Rotruck *et al.*, 1973).

GR activity was determined by using the method of Mohandas *et al.*, in which the following reaction is implicated:

NADPH + H^+^ + GSSG → NADP^+^ + 2GSH

In the presence of GR, oxidized glutathione undergoes reduction and simultaneously NADPH is oxidized to NADP^+^. Enzyme activity is quantified at room temperture by measuring the disappearance of NADPH/min at 340 nm spectrophotometrically (Mohandas *et al.*, 1984).

### Histopathological studies

The parts of the livers which were stored in 10% (w/v) buffered formalin were embedded in paraffin, sections cut at 5 μm and stained with hematoxylin and eosin (H/E). These sections were then examined under a light microscope for histo-architectural changes.

### Statistical analysis

The results of hepatoprotective and antioxidant activities are expressed as mean ± SEM. Results were statistically analyzed using one-way ANOVA, followed by the Tukey–Kramer post test for individual comparisons. The *p<*0.05 was considered to be significant.

## Results

### Biochemical parameters

**Figure 1:**
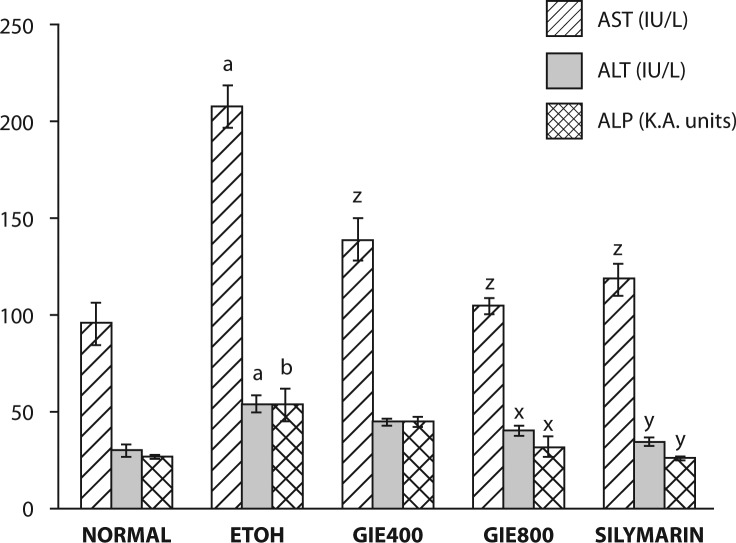
Effect of *Garcinia indica* extract on serum marker enzymes in ethanol intoxicated rats. *p-*values: ^a^<0.001, ^b^<0.01 when ETOH Control compared with Normal Control; ^x^<0.05, ^y^<0.01, ^z^<0.001 when Experimental groups compared with ETOH Control.

**Figure 2:**
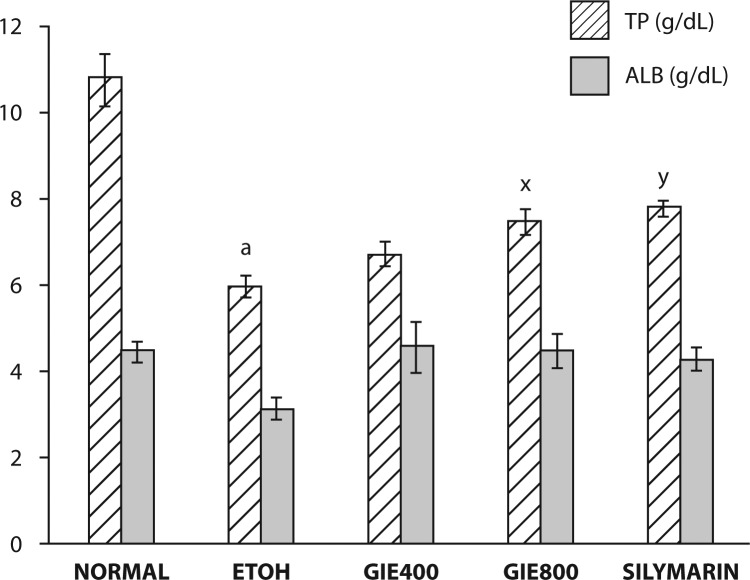
Effect of *Garcinia indica* extract on serum albumin and total proteins in ethanol intoxicated rats. *p-*values: ^a^<0.001 when ETOH Control compared with Normal Control; ^x^<0.05, ^y^<0.01 when Experimental groups compared with ETOH Control.

**Figure 3:**
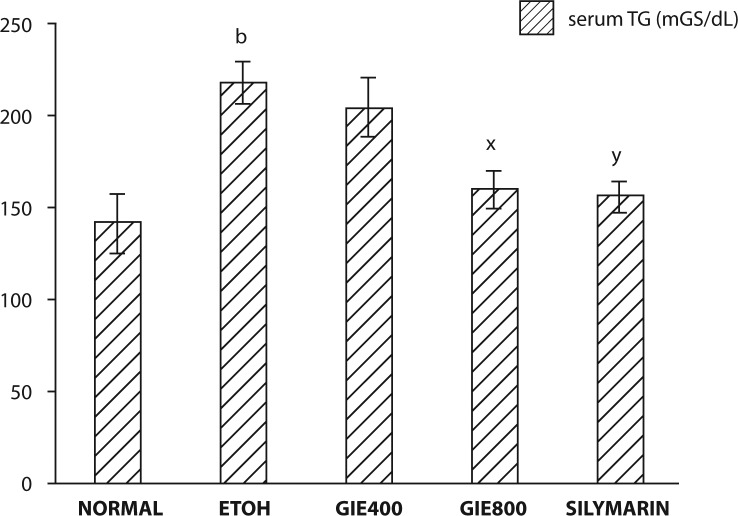
Effect of *Garcinia indica* extract on serum triglycerides in ethanol intoxicated rats. *p-*values: ^b^<0.01 when ETOH Control compared with Normal Control; ^x^<0.05, ^y^<0.01 when Experimental groups compared with ETOH Control.

The effects of GIE on serum marker enzymes AST, ALT and ALP and serum Alb, TP and serum triglycerides are summarized in Figures 1–3. Significant elevation in the marker enzyme activities and triglyceride levels, and depletion in Alb and TP content was observed in the ETOH–treated group of animals when compared with normal animals. Treatment of GIE400, GIE800 and silymarin to rats intoxicated with ETOH decreased significantly (*p<*0.001) the elevated activity of AST. However, ALT and AST activities were significantly decreased only by GIE800 and silymarin administration (*p<*0.05 and *p<*0.01 respectively). ETOH treatment significantly (*p<*0.001) decreased serum TP levels which were restored by GIE800 and silymarin significantly (*p<*0.05 and *p<*0.01 respectively) and the Alb levels were restored by treatment with GIE800, GIE400 and silymarin insignificantly. The serum triglyceride levels elevated by ETOH treatment were significantly restored (*p<*0.05) by GIE800 treatment, which was comparable to the effect of silymarin.

The effects of GIE on antioxidant enzymes, GSH, LPO and hTG are summarized in Table 1. MDA, the lipid peroxidation marker was significantly elevated (*p<*0.01) in the ETOH control group of rats when compared with the normal group. Treatment with GIE800 and silymarin to ETOH-intoxicated rats increased significantly (*p<*0.05 and *p<*0.01 respectively) the decreased levels of GSH, while GIE400 restored the GSH levels insignificantly.

**Table 1:** Effect of GIE on liver TBARS, GSH, SOD, CAT, GPx, GR and hTG in ETOH- intoxicated rats.

Biochemical parameters	Group I Normal Control distilled water	Group II Toxicant Control ETOH (5 g/kg, 20% w/v)	Group III GIE (400 mg/kg)	Group IV GIE (800 mg/kg)	Group V Silymarin (200 mg/kg)
TBARS (nmol MDA/g wet tissue)	34.72±4.07	59.31±7.32^b^	50.69±1.01	39.86±1.62^x^	34.89±1.77^y^
GSH (mol/g wet tissue)	1.33±0.03	1.04±0.02^a^	1.08±0.01	1.15±0.02^x^	1.19±0.02^y^
SOD (U/mg protein)	43.57±0.86	32.60±1.55^a^	36.61±2.35	42.23±1.81^y^	43.55±0.84^z^
CAT (U/mg protein)	5.13±1.05	1.50±0.28^b^	3.11±0.11	4.69±0.37^y^	5.02±0.71^y^
GPx (U/mg protein)	2.5±0.32	1.00±0.27^a^	1.96±0.06^x^	2.28±0.13^y^	2.4±0.19^y^
GR (U/mg protein)	798.32±35.44	256.15±61.89^a^	437.38±28.30	467.03±75.98^x^	576.25±31.58^y^
HTG (mg/dL)	2.37±0.30	13.21±1.01^a^	6.07±0.57^z^	4.40±0.24^z^	2.85±0.33^z^

Values are mean ± SEM for 6 animals in each group

a *p*-values: <0.001

b <0.01

c <0.05 when Toxicant Control compared with Normal Control

x <0.05

y <0.01

z <0.001 when Experimental groups compared with Toxicant Control

1 unit of CAT = µmol H_2_O_2_ consumed / min / mg protein

1 unit of GPx = µg GSH utilized / min / mg protein

1 unit of GR = nmol NADPH oxidized / min / mg protein

A significant decline in GSH levels (*p<*0.001) was observed in the ETOH treated group when compared with the normal control group of rats. Treatment with GIE800 and silymarin to ETOH-intoxicated rats increased significantly (*p<*0.05 and *p<*0.01 respectively) the decreased levels of GSH, while GIE400 restored the ETOH elevated levels insignificantly.

Liver SOD activity was found to be strikingly lower (*p<*0.001) in ETOH treated rats when compared with normal rats. Treatment with GIE800 and silymarin significantly (*p<*0.01 and *p<*0.001 respectively) restored SOD activity to normal, while GIE400 restored SOD activity insignificantly.

The CAT activity in livers of the ETOH treated group was found to be significantly (*p<*0.01) lower than in the normal group. GIE800 and silymarin treatments increased the ETOH-depleted CAT activity significantly (*p<*0.01).

GPx activity was depleted significantly (*p<*0.001) by ETOH treatment. Treatment with GIE400, GIE 800 and silymarin restored significantly (*p<*0.05, *p<*0.01 and *p<*0.01 respectively) the ETOH-depleted GPx activity.

GR activity was depleted significantly (*p<*0.001) by ETOH treatment. Treatment with GIE800 and silymarin restored significantly (*p<*0.05 and *p<*0.01 respectively) the ETOH-depleted GR activity.

Hepatic triglyceride levels were significantly elevated (*p<*0.001) in the ETOH control group of rats when compared with the normal group. Treatment of GIE400, GIE800 and silymarin to ETOH-intoxicated rats depleted hepatic triglyceride levels significantly (*p<*0.001).

### Histopathological studies

**Figure 4:**
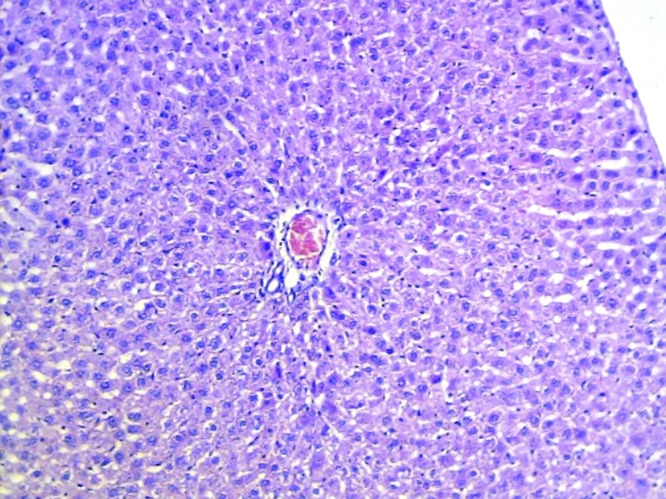
H/E staining of liver tissue of normal rats (100×).

**Figure 5:**
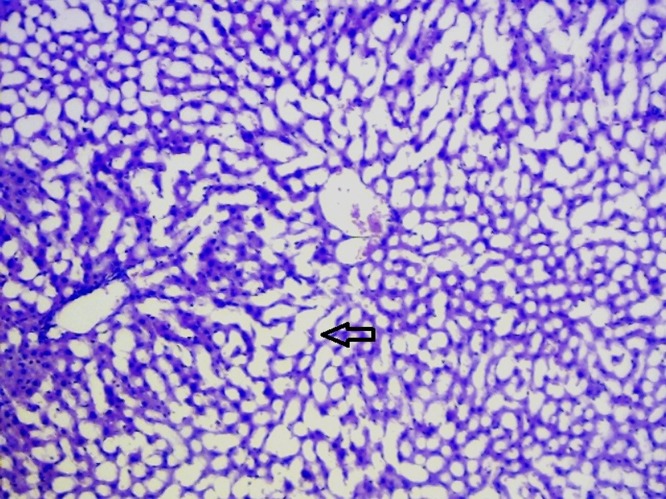
H/E staining of liver tissue of ethanol treated rats (100×).

**Figure 6:**
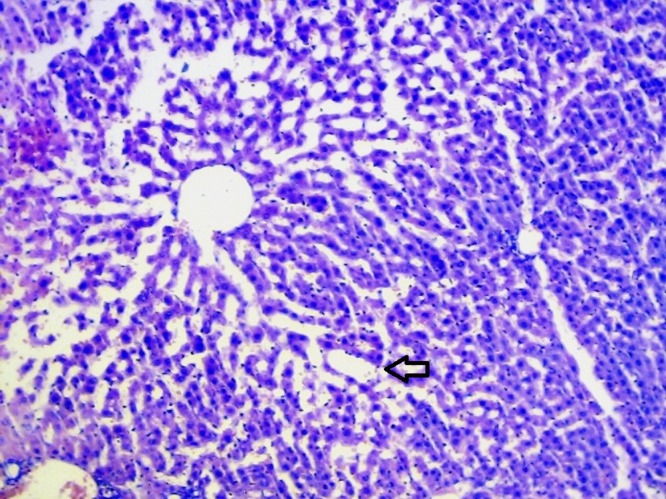
H/E staining of liver tissue of rats treated with ethanol and silymarin (200mg/kg) (100×).

**Figure 7:**
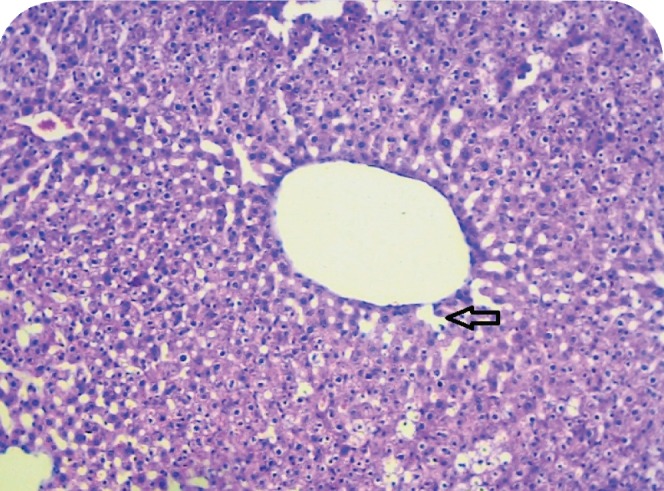
H/E staining of liver tissue of rats treated with ethanol and *Garcinia indica* extract (400 mg/kg) (100×).

**Figure 8:**
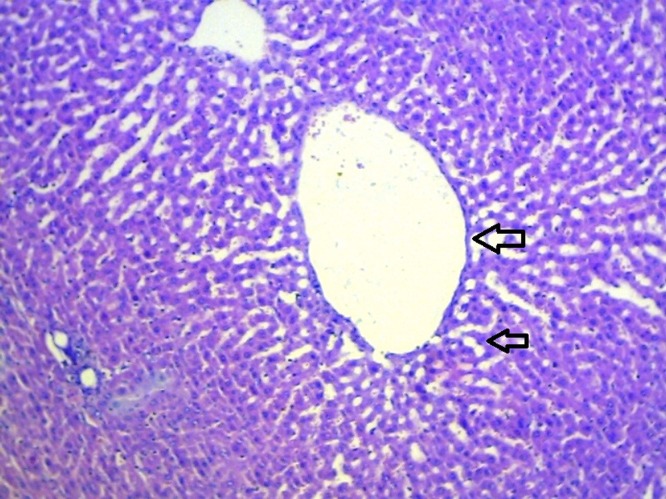
H/E staining of liver tissue of rats treated with ethanol and *Garcinia indica* extract (800 mg/kg) (100×).

The livers of animals of the normal group showed normal histology (Figure 4). The liver sections of animals treated with ETOH (Figure 5) showed a moderate to marked degree of fatty changes. There were moderate to severe abscesses and hemorrhage. The central veins showed congestion with congestion of sinusoids. Many hepatocytes showed degenerative changes. Moderate degree of mononuclear inflammatory infiltration was seen within all zones. Compared with the lesions observed in the ETOH group, the lesions noted in livers of silymarin treated animals were of a much milder degree (Figure 6). Few of the central veins and sinusoids showed dilatation with focal congestion and minimal fatty changes. Mild stromal inflammatory infiltration comprising lymphocytes and macrophages were seen also within the periportal and focal midzonal areas. Regenerative foci of mild to moderate degree were noted in these areas. The livers in the GIE400 group (Figure 7) showed mild to moderate degree of mononuclear inflammatory infiltration within all zones. Moderate fatty changes and few regenerative cells were noted. The GIE800 group (Figure 8) showed mild to moderate degree of mononuclear inflammatory infiltrations within all zones. Mild to minimal fatty changes around the dilated central vein were seen and many regenerative cells were noted. Histo-architecture of GIE800 treated rats tended to be more normal compared to GIE400, while silymarin treated rats showed almost normal histology.

## Discussion

The close relation between ethanol and liver damage is mainly due to the fact that about 80% of ingested alcohol is metabolized in the liver. Ethanol is metabolized into cytotoxic acetaldehyde by the enzyme alcohol dehydrogenase in the liver. Acetaldehyde is oxidized to acetate by aldehyde oxidase or xanthine oxidase, giving rise to various ROS via cytochrome P450 2E1 (Tuma & Casey, 2003). Alcohol damages also the mitochondria, resulting in decreased ATP production (Thurman *et al.*, 1984a). In addition, alcohol depletes the endogenous antioxidants and thereby, induces oxygen deficiency (hypoxia) creating a state of oxidative stress (Thurman *et al.*, 1984a; b).

Assessment of liver function can be performed by determining the activity of serum enzymes AST, ALT and ALP, originally present in high concentrations in the cytoplasm. When there is hepatic injury, these enzymes leak into the blood stream in conformity with the extent of liver damage. The elevated activities of these marker enzymes in sera of the ETOH-treated rats in the present study were due to the extensive liver damage caused by the toxin. Treatment with the test drug GIE (in both doses) as well as the reference drug silymarin significantly reduced the ETOH induced elevation in the activities of these enzymes.

Triglycerides are an important part of the cholesterol profile that is often measured quantitatively for diagnosis of primary and secondary hyperlipoproteinemia, dyslipidemia, triglyceridemia and liver obstruction. Results of the present study revealed increased serum triglyceride levels in ethanol fed rats, suggestive of decreased lipoprotein lipase enzyme activity, which is responsible for uptake of lipoprotein by extra-hepatic tissues. Decreased hepatic triglyceride level in GIE treated rats reflects the protective hepatocellular effect. According to the findings of Walker and Gordan, fatty infiltration of the liver occurs in rats after prolonged ingestion of ethanol (Walker & Gordon, 1970). Alterations in lipid metabolism brought about by ethanol can produce decreased oxidation, increased hepatic fatty acid synthesis, decreased triglyceride conversion and marked hepatic triglyceride accumulation (Walker & Gordon, 1970; Johnson, 1995).

Ethanol aggravates lipid peroxidation, as evident from the present study. Oral administration of GIE decreased the lipid peroxidation of cellular memberane, suggesting a protective effect. The increase in MDA levels in the livers of ETOH treated rats suggests enhanced peroxidation leading to tissue damage and failure of the antioxidant defense mechanisms to prevent the formation of excessive free radicals (Naik, 2003). Treatment with GIE significantly reversed these changes. It is thus likely that the mechanism of hepatoprotection of GIE lies in its antioxidant activity.

Reduced glutathione is one of the most abundant non-enzymatic antioxidant bio-molecules present in tissues (Meister, 1984). Its functions are removal of free oxygen species, such as H_2_O_2,_ superoxide anions & alkoxy radicals, maintenance of membrane protein thiols, and it acts as a substrate for GPx and glutathione *S*-transferase (GST) (Townsend *et al.*, 2003). In the present study, decreased GSH levels in ETOH intoxicated rats may have been caused by its increased utilization for augmenting the activities of GPx & GST and for detoxification of acetaldehyde during chronic ethanol exposure. GSH levels depleted by ETOH were significantly restored by GIE, either due to increased synthesis of GSH or due to stimulation of GR activity.

Free radical scavenging enzymes, such as SOD, CAT & GPx, are known to be the first line cellular defense enzymes against oxidative damage, disposing O_2_ & H_2_O_2_ before their interaction to form the more harmful hydroxyl (OH^..^) radical (Ji *et al.*, 1988). In the present study, SOD activity decreased significantly in the ETOH treated group of animals, which might be due to an excessive formation of superoxide anions. These excessive superoxide anions might inactivate SOD and decrease its activity. In the absence of adequate SOD activity, superoxide anions are not dismuted into H_2_O_2,_ which is the substrate for the H_2_O_2_ scavenging enzymes CAT & GPx. This results in inactivation of the H_2_O_2_ scavenging enzymes CAT and GPx, leading to a decrease in their activities. Administration of GIE to ETOH intoxicated rats effectively prevented the depletion of SOD, CAT & GPx activities, which can be correlated to the scavenging of free radicals by GIE, resulting in protection of these enzymes.

GR is an antioxidant enzyme involved in the reduction of GSSG (an endproduct of GPx reaction) to GSH. In ETOH treated rats, there was a marked depletion of GPx activity, leading to reduced availability of the substrate for GR, thereby decreasing the activity of GR. Oral treatment of GIE to ETOH intoxicated rats restored the activity of GR, thus accelerating the conversion of GSSG to GSH and enhancing the detoxification of reactive metabolites by conjugation with GSH.

The hepatoprotective effect of GIE may thus be attributed to an underlying antioxidant activity, which prevents the process of initiation and progress of hepatocellular injury. As confirmed by histopathological studies, the effects of GIE were comparable with those of silymarin, a proven hepatoprotective.

In conclusion, the hepatoprotective effect of GIE against ETOH-induced hepatotoxicity in rats appears to be related to the inhibition of lipid peroxidative processes and to the prevention of GSH depletion. The present study highlighted the health benefits of kokum, establishing it as a potent “functional food” and promoting its use as a culinary spice to enrich people's diets. Moreover, it is cheap, readily available to all strata of society, with medicinal properties attributed to it.
